# Modulation of microglial function by the antidepressant drug venlafaxine

**DOI:** 10.2478/intox-2014-0029

**Published:** 2015-03-04

**Authors:** Michal Dubovický, Eszter Császár, Kristína Melicherčíková, Marcela Kuniaková, Lucia Račková

**Affiliations:** 1 Institute of Experimental Pharmacology & Toxicology, Slovak Academy of Sciences, Department of Developmental and Behavioral Toxicology, Bratislava, Slovakia; 2 Institute of Medical Biology, Genetics and Clinical Genetics, Faculty of Medicine, Comenius University in Bratislava, Bratislava, Slovak Republic

**Keywords:** venlafaxine, microglia, inflammation, cytoprotection, depression

## Abstract

An increasing amount of data suggests that depression is an inflammatory disease. Depressed patients have higher peripheral blood levels of inflammatory markers which have been shown to access the brain and interact with the pathophysiological domain known to be involved in depression. Furthermore, microglia activation may play an important role in the inflammatory pathophysiology of depression.

In BV-2 microglia cell line, the present study investigated the potential anti-inflammatory effects of venlafaxine, along with its potential influence on injury of lipopolysaccharide (LPS)-stimulated cells. Although venlafaxine showed only marginal influence on the majority of the pro-inflammatory parameters assessed (in particular NO release, phagocytosis and proliferation), it significantly suppressed superoxide production by the stimulated cells. In addition, venlafaxine exerted also a protective effect on mitochondrial membrane potential and lysosomes of the stimulated microglia.

In conclusion, our results suggest that although VEN might have only a marginal effect on major pro-inflammatory parameters of microglia, its inhibitory effect on superoxide generation can contribute to the prevention of harmful effects of oxidative and nitrosative stress involved in the pathogenesis of depression. Moreover, the protective effect of VEN on viability of microglia can prevent a rapid reduction of these cells, thus avoiding limitations of several physiological processes in the brain and possibly also the progression of depression.

## Introduction

Depression, induced mostly by the hectic way of life and a high level of everyday stress, is a growing health concern in many countries. Although depression as a biochemical phenomenon was recognized in the mid-60s of the 20th century (Schildkraut, 1965), the etiology of the disease from a biological point of view is still not fully understood. According to previous studies, the background of depression is multifactorial, including reduced levels of neurotransmitters, hormone dysregulation, altered neuroplasticity, genetic factors as well as macroscopic changes of the brain (Brummelte *et al.*, 2012). However, none of these hypotheses does fully explain the whole symptomatology observed in this human condition.

The treatment of depression has so far been focused on stabilization of the neurotransmitter systems (Brummelte, *et al.*, 2012). According to the monoamine hypothesis, there is a functional deficiency of monoamines as serotonin, noradrenaline and dopamine in the brain. Specific manifestations of depression are due to the decreased level and the reduced density of specific receptors for each monoamine in the brain (Meyer *et al.*, 2006). Any change at the level of neurotransmitters, such as serotonin and catecholamines, has a strong influence on behavior and can result in typical manifestations of depressive behavior (Nestler *et al.*, 2008).

However, increasing amounts of data suggest that depression is an inflammatory disease (Raison *et al.*, 2006; Hashioka *et al.*, 2007; Tynan *et al.*, 2012; Obuchowicz *et al.*, 2014). Depressed patients have increased levels of pro-inflammatory cytokines in peripheral blood, acute phase proteins and chemokines such as IL-1β, IL-2, IL-6, IFN-γ, TNF-α, IL-6 receptor and IL-1 receptor, which have been shown to access the brain (Levine *et al.*, 1999; Lanquillon *et al.*, 2000; Miller *et al.*, 2009; Tynan *et al.*, 2012; Young *et al.*, 2014). Proinflammatory cytokines have been found to interact with many of the pathophysiological domains that characterize depression, including neurotransmitter metabolism, neuroendocrine function, synaptic plasticity and behaviour (Miller *et al.*, 2009). Cytokine-induced behavioural changes are associated mainly with alterations in the metabolism of serotonin, norepinephrine and dopamine in brain regions essential to the regulation of emotion, including the limbic system (amygdala, hippocampus and nucleus accumbens), as well as the regulation of psychomotor function and reward, including basal ganglia (Dunn *et al.*, 1999; Hamidi *et al.* 2004; Raison *et al.*, 2006; Goshen *et al.*, 2008). Futhermore, pro-inflammatory cytokines can lead to a reduction in the synthesis of 5-hydroxytryptamine (5-HT) and to increased levels of the neurotoxic metabolites by promoting the conversion of the 5-HT precursor tryptophan to kynurenine (Massart *et al.*, 2012), which may cause neuronal apoptosis and damage of glial cells (Maletic *et al.*, 2007). Depression can also promote inflammatory responses through effects on sympathetic and parasympathetic nervous system pathways.

The association between depression and inflammation is evident even in the context of mild depressive symptoms that do not meet criteria for major depression (Raison *et al.*, 2006, Miller *et al.*, 2009; Horikawa *et al.*, 2010; Tynan *et al.*, 2012; Obuchowicz *et al.*, 2014). Even single depression-related symptoms – such as fatigue, insomnia – have been associated with evidence of inflammatory activation in otherwise healthy individuals (Suarez *et al.*, 2002; Suarez *et al.*, 2004; Raison *et al.*, 2006)

Increasing evidence suggests that microglial activation may play an important role in the inflammatory pathophysiology of depression (Henry *et al.*, 2008; Steiner *et al.*, 2008; Tynan *et al.*, 2010). Microglial cells are the main innate immune cells of the brain. These cells respond quickly to pathogens and injury, they accumulate in regions of degeneration and produce a wide variety of pro-inflammatory mediators and reactive oxygen and nitrogen species, which can be toxic to neurons.

Recent data showed that chronic psychological stress (a well-known contributor to the development of depression) increased activation of microglia in the prefrontal cortex of rats (Pan *et al.*, 2014). On the other hand, the anti-inflammatory drug minocycline was able to reverse both microglial dysregulation and improve cognitive dysfunction in stressed animals (Hinwood *et al.*, 2012). Chronic psychological stress was also shown to increase the presence of deramified microglia in the medial amygdala, prefrontal cortex, and hippocampus. In addition, isolated microglia produced markedly higher levels of IL-6, TNF-α, and MCP-1 after stimulation with LPS compared with microglia from control mice (Wohleb *et al.*, 2011). Elevated levels of these pro-inflammatory cytokines in the brain could contribute to the development and maintenance of depression (Dowlati *et al.*, 2010; Howren *et al.*, 2009). A persistently activated microglial phenotype in the hippocampus and prefrontal cortex followed by enhanced depression-like behavior was seen in mice deficient in the fractalkine receptor (CX3CR1) in microglia after treatment with LPS (Corona *et al.*, 2010). Moreover, indoleamine 2,3-dioxygenase (IDO) and kynurenine monooxygenase (KMO), enzymes responsible for generation of neurotoxic tryptophane metabolites, were also increased in microglia from CX3CR1 knockout mice (Alboni *et al.*, 2010). On the other hand, previous studies showed that overactivation of microglia might actually be injurious to themselves and can eventually induce apoptotic cell death (Liu *et al.*, 2001). This might be an essential self-regulatory mechanism governing immune and inflammatory responses in the brain. However, with regard to a relatively limited capacity of the brain to resupply microglia (Cuadros & Navascués, 1998; Thomas, 1999), it has been suggested that premature depletion of these cells due to overactivation may handicap certain physiological processes in the brain, such as innate immune defence or protein homeostasis maintenance. Moreover, recent data showed that exposure of mice to a period of chronic unpredictable stress (CUS) induced apoptosis of microglia in the hippocampus due to overactivation followed by suppression of neurogenesis and appearance of depressive-like behavior (Kreisel *et al.*, 2014). On the other hand, the agents stimulating microglial proliferation reversed the depressive-like behavior and increased hippocampal neurogenesis.

The selective serotonin reuptake inhibitor (SSRI) and the serotonin noradrenalin reuptake inhibitor (SNRI) are commonly prescribed for treating major depression. It is well known that SSRIs and SNRIs work by inhibiting the reuptake of serotonin, noradrenaline and dopamine, resulting in an increase in the extracellular concentrations of these monoamines and thus in an increase in their neurotransmission. SSRIs and SNRIs are well tolerated with few side effects. However, several antidepressants were also found to inhibit the neurotoxic activation of microglia induced by LPS and cytokines in vitro (Obuchowicz *et al.*, 2006; Hashioka *et al.*, 2007; Chang *et al.*, 2009). This effect has been shown for different classes of antidepressants, including SSRI, SNRI, tricyclic antidepressants, and even ketamine.

Although some mild anti-inflammatory effects of venlafaxine have been already reported (Tynan *et al.*, 2012), a more detailed characterization of its anti-inflammatory profile is still needed. In addition, in view of recent studies, the possible effect of venlafaxine on activation-induced microglia injury should be also elucidated.

Therefore, in this study, we investigated the potential anti-inflammatory effects of venlafaxine in BV-2 microglia cell line and assessed also its potential influence on injury of lipopolysaccharide (LPS)-stimulated microglia.

## Methods

### Chemicals

Thiazolyl blue tetrazolium bromide (MTT), acridine orange, naphthylethylenediamine dihydrochloride, sulfanilamide, polystyrene fluorescent yellow-green latex beads, 2-(4-amidinophenyl)-6-indolecarbamidine dihydrochloride (DAPI), nitrotetrazolium blue chloride (NBT) and other chemicals were obtained from Sigma (Bratislava, Slovakia), unless otherwise stated. JC-1 was obtained from Santa Cruz Biotechnologies Inc. (Heidelberg, Germany). All reagents were of analytical grade or the highest possible purity. Venlafaxine was purchased from Chemoz, Czech Republic (purity, 98.5%).

### Cell culture and administration of the compounds tested

The immortalized mouse microglial cell line BV-2 (Biasi *et al.*, 1990) was cultured in DMEM, supplemented with 10% FBS (Biotech Ltd., Bratislava, Slovakia), and 1% P/S (100 U/ml penicillin, 100 µg/ml streptomycin; Biotech Ltd., Bratislava, Slovakia) and maintained in 5% CO_2_ at 37 °C. Cells were used for 10 passages at maximum. The stock solution of venlafaxine was prepared in distilled water. The inflammogen was added to the cells in the presence of the compound tested or of a vehicle.

### Viability assay

The cells were grown in 96-well microplates, in complete DMEM. At the end of the incubation with VEN, the cells were incubated with MTT (0.5 mg/ml) in DMEM in 5% CO_2_ at 37 °C for 120 min. Subsequently, the formazan crystals were solubilized by addition of 100 µL of 10% SDS in HCl (0.01N). The absorbance was spectrophotometrically recorded at 570 nm.

### Production of NO by BV-2 microglia

After treating the cells grown in 96-well plates with LPS in the presense or absence of VEN, nitrite was measured in culture supernatants to assess NO production in microglial cells using the Griess method (Griess, 1879). Briefly, sample aliquots (100 µl) were mixed with 100µl of Griess reagent (1% sulfanilamide/0.1% naphthylethylene diamine dihydrochloride/2% phosphoric acid) and incubated at 25 °C for 10 min. The absorbance at 540 nm was measured on a microplate reader.

### Quantification of superoxide by NBT assay (Rook *et al.*, 1985)

NBT solution was added per well to the adherent cell monolayers (final concentration 1 mg/ml) and the plate was incubated for further 1.5 h at 37 °C. At the end of incubation, the cells were washed carefully with methanol and the wells were then allowed to air-dry. The blue formazan was solubilized by adding first 60 µl/well 2 M KOH and then 70 µl/well DMSO, followed by thorough mixing. The absorbance was read on the plate reader at 630 nm.

### Phagocytosis assay

The cells were grown in 96-well plates and treated with LPS in the presence or absense of VEN. A suspension of carboxylate-modified polystyrene yellow-green latex beads (1 µm; Sigma) was added directly to the culture medium (0.05%) after 16 h of stimulation with LPS (1 µg/ml) and allowed to incubate for further 4 h. Then the cells were washed trice with PBS and the fluorescence was determined *in situ* at excitation 480 nm and emission 530 nm.

### Proliferation assay

The cells were grown in 96-well plates and treated with LPS in the presence or absence of VEN. The incubation medium was removed and the solution of DAPI (5µg/ml) in PBS was added to the cells. After 20 min incubation, the staining solution was removed, the cells were washed with PBS and the fluorescence was read at excitation 359 nm and emission 461 nm.

### Lysosomal stability assay

Lysosomal integrity was assessed as the acridine orange (AO) uptake assay (Persson *et al.*, 2003). The cells were grown in 96-well plates and treated with LPS in the presence or absence of VEN. Following treatment, the incubation medium was replaced with fresh medium containing AO solution (5µg/ml) and the cells were incubated in 5% CO_2_ atmosphere at 37°C for 20 min. After the AO solution was removed, the cells were washed twice with PBS and the fluorescence was read in a plate reader at excitation 488nm and emission 600 nm (red fluorescence) and at excitation 488nm and emission 525 nm (green fluorescence). The red to green fluorescence ratio was evaluated as the parameter of lysosomal integrity.

### Assessment of mitochondrial membrane potential

Following treatment, the incubation medium was replaced with freshly prepared JC-1 solution (5 µg/ml) in full DMEM in 5% CO_2_ atmosphere at 37°C for 30 min (Roy *et al.*, 2008). After JC-1 was removed, the cells were washed trice with PBS and the fluorescence was read in a plate reader at excitation 550 nm and emission 600 nm (red fluorescence) and at excitation 485 nm and emission 525 nm (green fluorescence). The red to green fluorescence ratio was evaluated as the parameter of mitochondrial membrane polarization.

### Statistical analysis

Data are presented as means ± standard deviation (SD) of three separate experiments. Statistical analysis was performed using Student′s t-test and statistical significance is expressed as *p<0.05; **p<0.01; ***p<0.001 *vs* control and ^&^ *p<*0.05; ^&&^ *p<*0.01; ^&&&^ *p<*0.001 *vs* LPS-treated cells.

## Results

### Cytotoxicity of venlafaxine on BV-2 microglia

**Figure 1:**
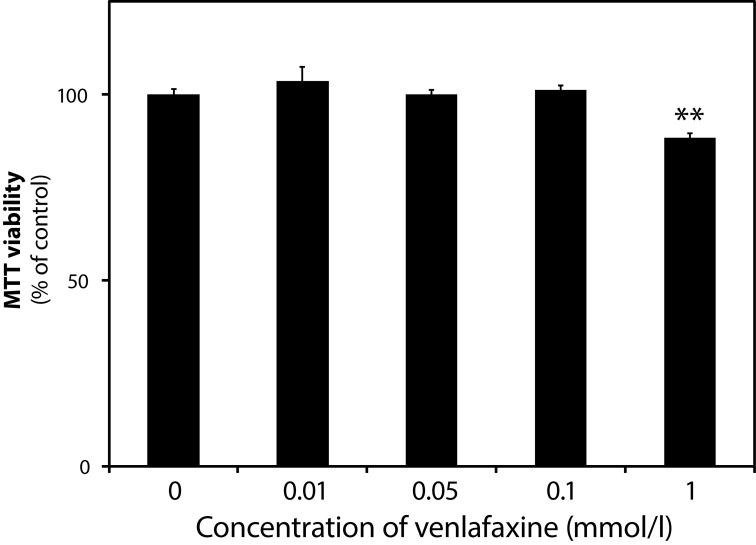
Viability of BV-2 microglial cells incubated for 24hrs with or without venlafaxine. Data are means ± SD from 3 independent experiments, **p<0.01 *vs* control.

As confirmed by the MTT assay, VEN did not affect the viability of BV-2 microglial cells in a concentration range used for further assays (Figure 1). Moreover, only a mild viability injury (88.3±1.22% of control, *p<*0.01) was observed at a high non-physiological concentration (1 mmol/l).

### NO production, phagocytosis and proliferation of BV-2 microglia

**Figure 2:**
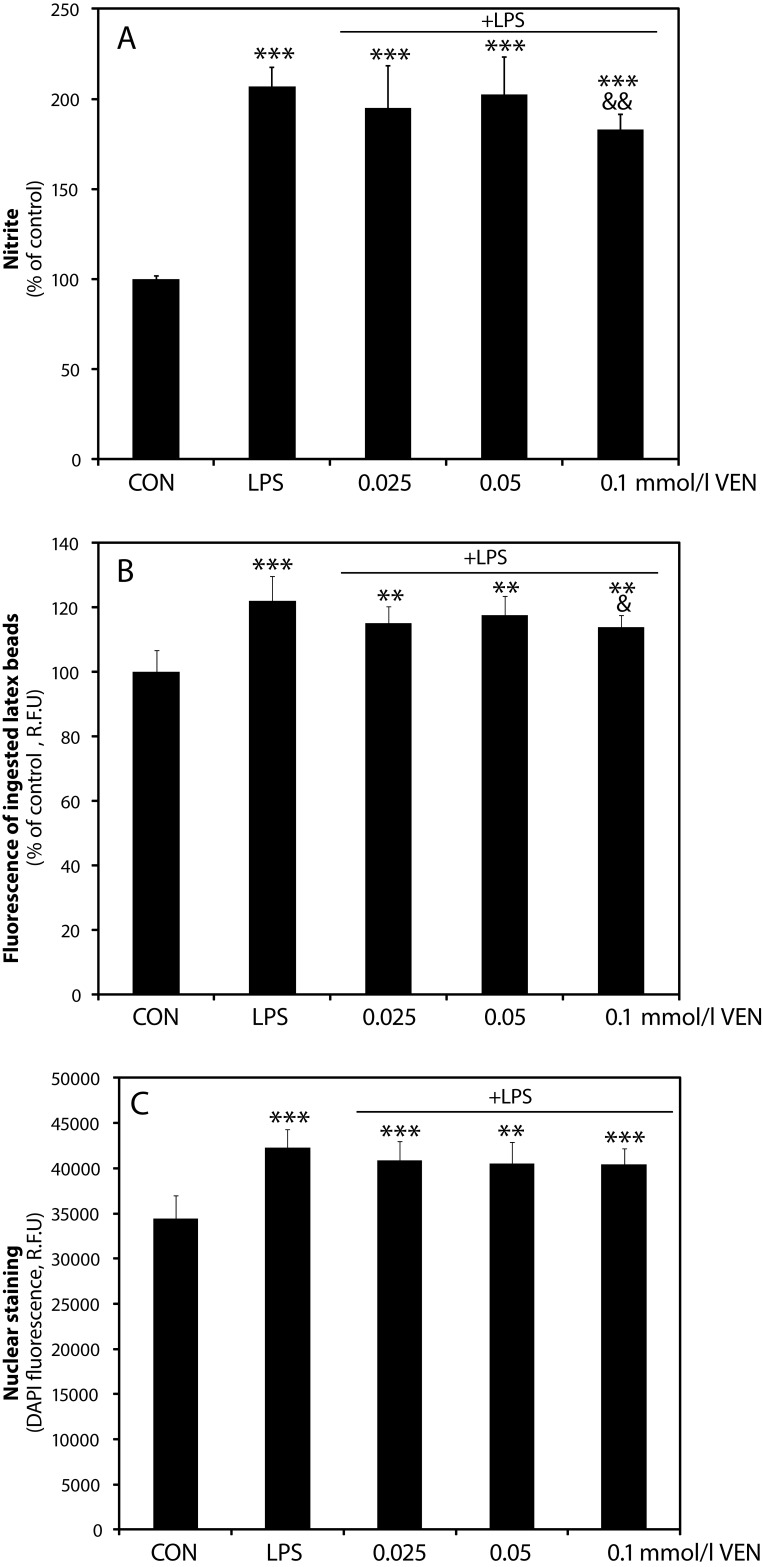
Effect of VEN on inflammatory markers of stimulated BV-2 microglia. Effect of VEN on A: NO production, B: fluorescent latex bead phagocytosis, and C: proliferation assessed by DAPI staining of BV-2 cells stimulated with LPS (1 µg/ml). BV-2 cells were treated with LPS in the presence or absence of VEN for 24 hours and then NO release into media was evaluated. Phagocytosis was assessed within 16 hours of stimulation in the presence or absence of VEN. CON - control; LPS – lipopolysaccharide; VEN – venlafaxine. **p<0.01; ***p<0.001 vs control; ^&&^ *p<*0.01, ^&^ *p<*0.05 vs LPS.

Venlafaxine showed only a mild inhibitory effect on nitric oxide (NO) production by BV-2 microglia stimulated with bacterial lipopolysaccharide (LPS, 1 µg/ml). A marginal inhibition of NO release was shown only at 100 µmol/l concentration of VEN (183±8.31%, *p<*0.01 *vs* LPS-stimulated cells, 207±10.6%, Figure 2A). The lower concentrations tested did not influence the NO levels in the media. Moreover, only at 100 µmol/l concentration did VEN mildly suppress phagocytosis of fluorescent latex beads (114±3.72%, *p<*0.05 *vs* LPS-stimulated cells, 122±7.66%, Figure 2B). Furthermore, as shown by DAPI staining method adjusted for plate reader format, VEN in the concentration range tested did not influence the proliferation of LPS-stimulated microglia (Figure 2C).

### Superoxide production

**Figure 3:**
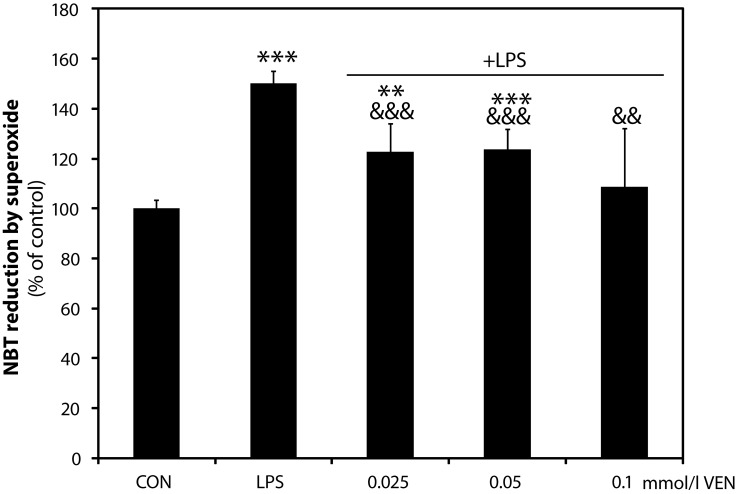
Effect of VEN on superoxide production in stimulated BV-2 microglia. Nitroblue tetrazolium salt conversion to formazan was assessed in BV-2 cells treated with LPS in the presence or absence of VEN for 16 hours. CON – control; LPS – lipopolysaccharide; VEN – venlafaxine. **p<0.01; ***p<0.001 vs control; ^&&^ *p<*0.01, ^&&&^ *p<*0.001 vs LPS.

At the concentration range tested, VEN significantly suppressed LPS-stimulated superoxide production, observed as a reduction of nitroblue tetrazolium salt, with the most profound inhibition at the maximum concentration tested (109±23.2%, *p<*0.01 at 100 µmol/l; 124±7.9%, *p<*0.001 at 50 µmol/l; 123±11.1%, *p<*0.01 at 25 µmol/l, *vs* LPS-stimulated cells, 150±5.0%, *p<*0.001; Figure 3).

### Mitochondrial membrane potential Δψ_m_ and lysosomal stability of BV-2 microglia

**Figure 4:**
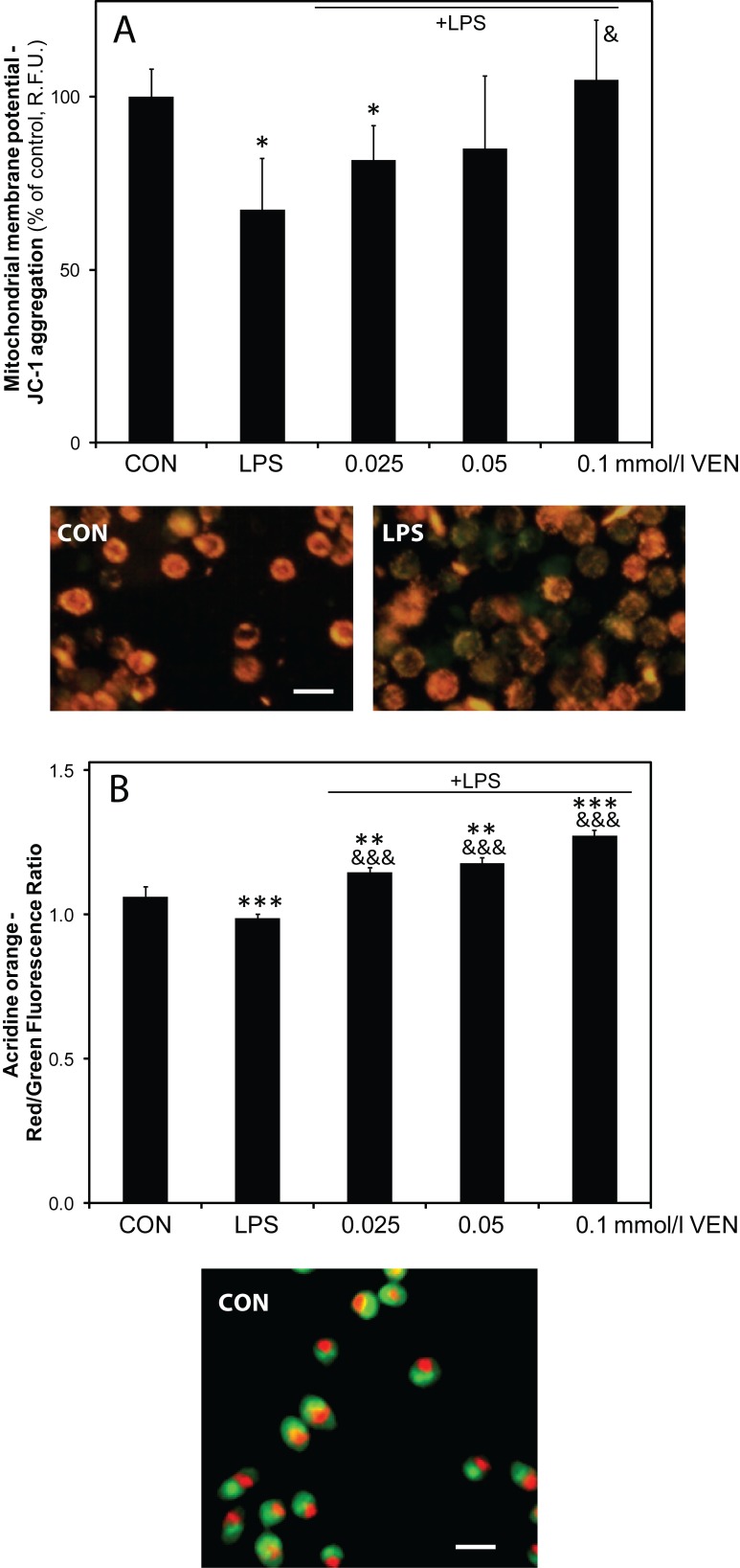
Effect of VEN on mitochondrial depolarization and lysosomal destabilization in stimulated BV-2 microglia. Effect of VEN on A: changes in mitochondrial membrane potential Δψ_m_ assessed as the red-to-green fluorescence ratio of the incorporated cationic dye JC-1; and on B: lysosomal stability assessed as the red-to-green fluorescence ratio of the incorporated cationic dye acridine orange. CON – control; LPS – lipopolysaccharide; VEN – venlafaxine. Scale bar: 20um. *p<0.05; **p<0.01; ***p<0.001 vs control; ^&^<0.05; ^&&&^p<0.001 vs LPS.

Stimulation with a higher concentration of LPS (10 µg/ml) caused a significant decrease of mitochondrial membrane potential in BV-2 cells (67.3±14.7% *vs* control cells, *p<*0.05), as documented by staining with the voltage-sensitive cationic dye JC-1 (emitting green fluorescence when the mitochondria are depolarized and red at intact Δψ_m_ when excited at 488 nm) (Figure 4A). Co-treatment with VEN increased the red fluorescence of JC-1 in the LPS-stimulated cells with a significant effect at the highest concentration tested (105±17.3%, *p<*0.05 *vs* LPS-stimulated cells at 100 µmol/l; 84.9±21.1%, *p>*0.05 *vs* LPS-stimulated cells at 50 µmol/l; 81.7±9.79%, *p>*0.05 *vs* LPS-stimulated cells at 25 µmol/l).

Stimulation with LPS (10µg/ml) caused also a mild destabilization of lysosomes, shown as a decrease of the red-to-green fluorescence ratio, corresponding to a decrease of the dye sequestration into the acidic organelles (Persson *et al.*, 2003). All the concentrations tested increased the red-to-green fluorescence ratio in comparison to either control or stimulated cells (120±1.92%, *p<*0.001 *vs* control cells and *p<*0.001 *vs* LPS-stimulated cells at 100 µmol/l; 111±1.92%, *p>*0.05 *vs* control cells and *p<*0.001 *vs* LPS-stimulated cells at 50 µmol/l; 108±1.41%, *p<*0.01 *vs* control cells and *p<*0.001 *vs* LPS-stimulated cells at 25 µmol/l).

## Discussion

Numerous studies have found that at the background of depression not only changes at the monoamine level but also alterations in the level of proinflammatory mediators come into play. Depressed patients showed significantly higher levels of peripheral blood inflammatory biomarkers, including inflammatory cytokines, such as IL-1, IL-2, lL-6, IFN-γ or TNF-α, than did healthy individuals. These biomarkers have been shown to access the brain and interact with the pathophysiologic domain known to be involved in depression (Miller *et al.* 2009).

Several studies have suggested an important role of Toll-like receptors (TLRs) in neuropathologies and neurodegeneration (Crack *et al.*, 2007). A large majority of the already discovered TLRs is expressed not only in immune cells but also in neurons, astrocytes and CNS-resident microglia. TLR-dependent microglia activation plays a crucial role in initiating host defence responses during microbial infection of the CNS. However, TLR4 activation in microglia with endogenous ligands, such as amyloid β were shown to be a critical mechanism also in the development of Alzheimer disease (Walter *et al.*, 2007). Moreover, data by Gárate *et al.* (2013) suggest that Toll-like receptor 4 (TLR-4) plays a critical regulatory role in the brain‘s response to stress. Thus psychological stress may compromise the intestinal barrier and increased gastrointestinal permeability with translocation of lipopolysaccharide (LPS) from Gram-negative bacteria may play a role in the pathophysiology of major depression (Gárate *et al.*, 2011).

Since alterations of monoamines are well controlled during depression with SSRI and SNRI medications, the question has been raised whether these antidepressants could have also anti-inflammatory effects. The anti-inflammatory character of SSRI and SNRI was initially related to work in the peripheral immune system, yet current evidence suggests that these drugs might exert an anti-inflammatory effect also on the brain-resident microglia. Most of the work in this field of research was done with SSRIs, while effects of SNRIs on microglia have not been clearly evaluated.

In the present study, using BV2 microglial cell line, we investigated if venlafaxine, a widely used antidepressant, could inhibit activation of microglia by bacterial LPS. In line with data reported by Tynan *et al.* (2012), VEN showed only marginal effect on activation markers of LPS-stimulated BV2 microglial cells, concerning particularly NO production and phagocytosis. In addition, VEN did not influence LPS-stimulated microglia proliferation. However, venlafaxine has been suggested to indirectly exert anti-inflammatory actions *in vivo* through elevation of norepinephrine, showing a potent anti-inflammatory effect (Dello Russo *et al.*, 2004).

Nevertheless, our data showed an inhibitory effect of VEN on superoxide generation. In view of the suggested role of oxidative and nitrosative stress in the pathogenesis of depression (Maes *et al.*, 2011), the superoxide scavenging effect might be an important therapeutic mechanism of VEN. A recent meta-analysis showed an association between depression and oxidative stress and antioxidant status across many different studies (Palta *et al.*, 2014). Furthermore, these findings suggest that well-established associations between depression and poor health outcomes may be mediated by high oxidative stress. On the other hand, a comprehensive overview over the current literature discussing the involvement of oxidative stress in depression showed that few of these reports were able to confirm correlations of reduced oxidative stress with antidepressant treatment (Michel *et al.*, 2012). In this regard, long-term treatment with VEN in the effective antidepressant doses was shown to protect against stress-induced oxidative cellular and DNA damage and promote antioxidant defence systems in the depressed animals (Eren *et al.*, 2007).

In addition, a crucial role for nitrosative stress in the pathophysiology of unipolar and bipolar depression was recently also reported (Moylan *et al.*, 2014). Peroxynitrite (ONOO^–^) is a highly reactive coupling product of nitric oxide and superoxide and it has been implicated in the pathogenesis of an increasing number of inflammatory diseases with possibly deleterious effects on host tissues. As shown by Zhou *et al.* (2011), peroxynitrite derived from hippocampal nNOS is a necessary signal for the role of glucocorticoids in mediating depressive behavior. In analogy with metoxy-phenol apocynin (Muijsers *et al.*, 2000), VEN downregulated stimulated superoxide production without remarkably influencing NO levels. However, as suggested by Muijsers *et al.* (2000), nitric oxide formation as such may not be essential for reactive nitrogen species (RNS)-mediated tissue damage during (neuro)inflammation. In contrast, superoxide and its secondary metabolites might have a fundamental role in all pathways leading to RNS formation. Consequently, in analogy with apocynin, limiting superoxide production by VEN may prevent the formation of peroxynitrite as well as other RNS, thus ameliorating concomitant tissue damage.

Contradictory to the viability data obtained by Tynan *et al.* (2012) using the Presto Blue assay, VEN did not reduce viability assessed as MTT reduction by mitochondrial dehydrogenases in our BV-2 cellular model. In support of our result, the maximum non-toxic concentration of VEN (100 µmol/l) tested significantly prevented LPS-induced depolarization of the mitochondrial membrane and showed also the most profound protective effect on lysosomal stability. As proposed by Brunk and Terman (2002), mitochondria and lysosomes may represent the critical organelles in various pathologies and in aging by retaining a vicious cycle of mutual toxic interactions leading eventually to the induction of cell death. Several antidepressant drugs were shown to accumulate in lysosomes (Daniel *et al.*, 2001) and occasionally, the lysosomal entrapment of a substance was shown to be associated with protection of these organelles through iron chelating effect (Persson *et al.*, 2003). Hence, VEN might potentially interfere with this vicious cycle and consequently prevent the induction of apoptosis in activated microglia. In view of the very recent data suggesting that overactivation-induced apoptosis of microglia in the hippocampus followed by suppression of neurogenesis may be a cause of the appearance of depressive-like manifestations in the chronically stressed animals (Kreisel *et al.*, 2014), it might be hypothesized that the protective effect of VEN on microglia viability could contribute to its anti-depressant mechanism.

In conclusion, our results suggest that although VEN might have but a marginal effect on major pro-inflammatory parameters of microglia, its inhibitory effect on superoxide generation can contribute to the prevention of harmful effects of oxidative and nitrosative stress involved in the pathogenesis of depression. Furthermore, the protective effect of VEN on viability of microglia can prevent rapid reduction of these cells, thus avoiding limitations of several physiological processes in the brain, and possibly also halt the progression of depression.
